# Therapist and treatment credibility in treatment outcomes: A systematic review and meta-analysis of clients’ perceptions in individual and face-to-face psychotherapies

**DOI:** 10.1080/10503307.2023.2298000

**Published:** 2024-01-04

**Authors:** Güler Beril Kumpasoğlu, Chloe Campbell, Rob Saunders, Peter Fonagy

**Affiliations:** 1 Research Department of Clinical, Educational and Health Psychology, University College London, London, UK; 2 Anna Freud National Centre for Children and Families, London, UK; 3 Department of Psychology, Ankara University, Ankara, Turkey

**Keywords:** treatment credibility, therapist credibility, treatment outcome, common factors, therapeutic relationship

## Abstract

**Objective:**

No systematic review was identified investigating the influence of perceived therapist credibility on treatment outcomes. Extant treatment credibility reviews have focused on early perceptions without considering influence of various therapy phases. This study aimed to examine the relationship between perceived treatment and therapist credibility and treatment outcomes, while considering the timing of the credibility assessment as a potential moderator.

**Method:**

Articles published in English peer-reviewed journals containing at least one quantitative measure of credibility and treatment outcome regarding face-to-face therapist-delivered interventions were eligible. PsycINFO, MEDLINE and Embase online databases were last searched on April 5th, 2023, and the Effective Public Health Practice Project tool was used to assess the study quality. Correlations between treatment credibility and outcomes, and therapist credibility were calculated separately.

**Results:**

Analysis of 27 studies revealed a positive association between perceived treatment credibility and treatment outcome (*r *= 0.15,95%CI = 0.09,0.21,*p *< 0.001,*n *= 2061). Nine studies showed a strong association between perceived therapist credibility and outcome (*r *= 0.35,95%CI = 0.18,0.51;*p *< .001*,n *= 1161). No significant moderator found in both meta-analyses.

**Conclusion:**

Findings suggest that clients’ perceptions of higher credibility – whether concerning the treatment or the therapist – are associated with better therapeutic outcomes. Constraints in inclusion criteria and the small sample size in eligible studies were notable limitations.

**Clinical or methodological significance of this article**: This study highlights the potential role of clients’ perceptions of the therapist and treatment credibility during both the initial and subsequent stages of psychotherapy in enhancing the efficacy of psychotherapy interventions. As the first study to meta-analytically review the effect of therapist credibility on treatment outcomes, the results of this review could inform the delivery of psychotherapies and have the potential to optimize treatment outcomes.

Common factors, which are variables that are not specific to any particular therapeutic approach but are nonetheless thought to influence psychotherapy outcomes (Cuijpers et al., 2019). The contextual model (Wampold, 2015), one of the explanatory models of the common factor theory, has emphasized the importance of the early perceptions of clients in the therapeutic process. During the initial sessions, clients make evaluative judgements about their therapist, such as whether they possess the necessary expertise, are trustworthy, and are willing to understand both the problem and its context (Wampold, 2015). Clients make similar evaluations about the form of treatment being offered, wherein the efficacy of the given treatment and its appropriateness for the client's problems are assessed (Coyne et al., 2019). Both evaluations converge on the construct of credibility, a well-known concept in the common factor theory; however, systematic reviews on this concept are limited.

Strong's (1968) model of social influence in counseling has provided an early theoretical basis for the potential role of therapist credibility in psychotherapy. According to Strong (1968), the counseling process is believed to be based on interpersonal influence, and its efficacy could be augmented by enhancing the “counselor's influence power over the client” (p. 223). Three factors of the counselor's potential social influence were identified: expertness, trustworthiness, and attractiveness, which were later combined into an overall concept of perceived credibility (Strong, 1968; Wolff & Hayes, 2009). Treatment credibility, on the other hand, refers to the client's perceptions of the believability, suitability, and “logicalness” of the treatment (Kazdin, 1979). While therapist and treatment credibility have often been considered conceptually aligned, there may be instances where a client believes in the efficacy of psychotherapy but questions the competence or reliability of the therapist, or vice versa (Constantino et al., 2018).

Research indicates that perceptions of both treatment and therapist credibility can change over the course of treatment, and that these differences in credibility assessment across time may be related to different therapeutic outcomes (Barnicot et al., 2016; Newman & Fisher, 2010). Accordingly, Hardy et al. (1995) provided an important distinction between initial and emergent treatment credibility. Initial credibility was conceptualized as the client’s perception of the treatment prior to the start of therapy, evaluated before the first session, and does not include the nuanced interpersonal dynamics of the therapeutic relationship between the client and therapist. It is believed to influence the client's decision to initiate or continue treatment (Hardy et al., 1995). Conversely, emergent treatment credibility encapsulates the perception of credibility after the client has experienced at least some treatment. This perception may be influenced by factors pertaining to the therapist, such as perceived expertise, as well as aspects related to the therapy process including engagement in treatment (Mooney et al., 2014). A reviewer highlighted that pre-treatment credibility might relate more to general perceptions about psychotherapy effectiveness, particularly when clients lack prior experience or specific information about the treatment.

The credibility of the treatment or therapist can be evaluated according to the ratings of the clients, independent evaluators, the client's significant others, or other therapists. Although variations were noted in the ratings of process variables among observers (Horvath & Symonds, 1991), clients’ ratings of the credibility have been suggested as a more robust predictor of therapy outcomes (Wolff & Hayes, 2009). Söchting et al. (2016), suggested that the strong interpersonal and social dynamics in group settings may diminish the influence of credibility. On the other hand, internet-based therapies are often perceived as less credible and less preferred than face-to-face therapies (Apolinário-Hagen et al., 2018). However, mixed-method studies, such as those by Wallin et al. (2016), revealed that the perceived lower credibility of internet therapies is not just due to therapy-related factors, like doubts about effectiveness, but also client-related factors, such as not taking online therapy seriously or struggling to find motivation. These findings indicated that different treatment modalities may influence how credibility is perceived by clients.

Various common factors concerning the client's assessments of the therapist and treatment have been explored in relation to credibility. One such factor is outcome expectancy, which refers to clients’ beliefs about the potential benefits of therapy (Kazdin, 1979). Although they are strongly correlated (Nock et al., 2007), outcome expectancy and treatment credibility may differ in their origins and likely trajectories (Thompson-Hollands et al., 2014; Uebelacker et al., 2018). Credibility perception is primarily formed through cognitive processes, whereas expectations are more closely linked to the client’s emotional processes (Devilly & Borkovec, 2000). Furthermore, expectancy includes clients’ ideas about their own issues and beliefs about the response of that particular problem to a specific type of therapy, that is, their optimism about recovery, and can be influenced by a client’s readiness to change (Harrison et al., 2019). Credibility, on the other hand, focuses on the therapeutic approach itself, such as the perceived logical coherence of the therapeutic model instead of making a determination about the likely outcomes of a treatment procedure for a specific person (Jacobson & Baucom, 1977). Another related concept to credibility is the working alliance; however, the working alliance is a broad and dynamic concept encompassing other aspects of the therapeutic relationship, including collaboration in treatment tasks (Bordin, 1979; Hatcher, 2021).

In summary, while the relationship between perceived credibility and outcomes in psychotherapy has been identified as an important factor in treatment effectiveness, systematic research examining this relationship is limited. We found no previous systematic reviews focused on the effect of therapist credibility on therapy results. However, therapist credibility may explain why certain therapists consistently attain better treatment outcomes compared to others, regardless of client characteristics or treatment type (Wampold, 2015). Regarding treatment credibility, a previous meta-analysis (Constantino et al., 2018) revealed a small but significant association between treatment credibility and psychotherapy outcomes. Nevertheless, the meta-analysis was limited in its scope, as it narrowly focused on early credibility perceptions (*pre-treatment, sessions 1 and 2*), disregarding any further evaluations. Furthermore, it amalgamated these perceptions into an “early credibility” category, neglecting the potential variability in credibility perceptions across distinct stages of treatment and their clinical implications. However, once therapy commences, the perceived treatment credibility might be influenced by interpersonal variables in the therapeutic relationship (Dryden & Sabelus, 2012). Moreover, aspects related to the treatment process such as early symptom improvement have been found to be associated with the perception of treatment credibility even as early as session 2 (Mooney et al., 2014). Furthermore, distinguishing between initial (i.e., pre-treatment) and emergent credibility perceptions may provide insights into potential clinical applications including the role of pre-treatment client characteristics in shaping credibility perceptions (Constantino et al., 2014). Hence, further research is needed to better explore the dynamic relationship between clients’ credibility perceptions and psychotherapy outcomes.

The aim of the present systematic review and meta-analysis was to address the existing gap in the literature by examining the correlation between clients’ perceptions of their therapist and treatment credibility with treatment outcomes in individual face-to-face psychotherapies. This study encompassed credibility assessment at any point during the therapeutic process and explore potential variations in outcome-credibility association throughout the treatment as well as at various stages of the treatment process, with particular emphasis on initial and emergent assessments.

## Methods

### Search Procedure

For the current systematic review, the Preferred Reporting Items for Systematic Reviews and Meta-analyses (PRISMA; Page et al., 2021) guidelines were followed (see Supplementary Material for PRISMA checklist). The review protocol was registered with the International Prospective Register of Systematic Reviews (PROSPERO), and subsequently amended to include meta-analytic data synthesis strategy. The registration number is *CRD42021243661*. An initial scoping review was conducted to inform the selection of search terms and systematic literature searches conducted in PsycINFO, MEDLINE, and Embase online databases using the OVID platform in March 2021, with an update search conducted on 5th April 2023. Additionally, reference lists of relevant empirical studies and reviews, and grey literature were checked, and a manual search was performed on Google Scholar (see Supplementary Material 1 for details of the search strategy). The first author removed duplicates and screened the titles and/or abstracts of each paper for eligibility. A sample (10%) of random articles was screened by the second author, both in the abstract/title screening stage and later at the full-text screening. When there was uncertainty about the eligibility of a paper, all authors discussed the relevance of the paper and made a decision accordingly.

### Inclusion and Exclusion Criteria

The review included empirical studies published between 1960 and 2023 in peer-reviewed journals written in English. The eligibility criteria were defined according to the PICO (Population/Patient, Intervention, Control/Comparison, and Outcomes) format: the population was comprised of adults or adolescents from any background who received *bona fide* individual therapist-delivered face-to-face psychotherapy, while self-help interventions, group therapies, couple therapies, and online or chat-based therapies were excluded. Treatments that were provided by trained therapists, based on established psychological theories, and recognized as a viable form of psychotherapy were considered *bona fide* (Wampold et al., 1997). Outcome was assessed using at least one quantitative assessment tool, and studies that assessed client perception of the therapist or treatment credibility using at least one quantitative measure were included. Control groups were not relevant to the review.

To minimize confounding variables, only studies which utilized face-to-face, therapist-delivered individual therapies were included. Studies that measured the credibility of the treatment or therapist by someone other than the client, such as a spouse, parent, caregiver, or interviewer were excluded. However, it should be noted that in certain papers, treatment credibility might have been conflated with treatment expectations; this is because almost all studies that have focused on treatment credibility utilized variations of the Credibility/ Expectancy Questionnaire (CEQ; Borkovec & Nau, 1972). However, there is variability across studies in the number of items employed from the credibility subscale. The most commonly utilized version includes three items: “How logical does the offered therapy appear to you?”, “How effective do you think this treatment will be in reducing your symptoms?”, and “How confident are you in recommending this treatment to a friend facing similar difficulties?” (Devilly & Borkovec, 2000). However, some studies included an additional item from the expectancy subscale, “By the end of the therapy period, how much improvement in your symptoms do you think will occur?”, and suggested that items with “*I think*” statements should be considered within the credibility subscale, whereas items with “*I feel*” should be classified as treatment expectation (Nock et al., 2007; Silva et al., 2021). Results from previous studies exploring the factor structure of the CEQ have been inconsistent. While some studies suggest that the 3-item subscale is the most reliable model (Coste et al., 2020), other studies proposed that the additional item had a cross-loading on both factors (Nock et al., 2007). Consequently, the utilization of items in the assessment of treatment credibility varied among the eligible papers. Therefore, as conducted by Constantino et al. (2018), only those studies that clearly identified that their predictor variable was treatment credibility were included in the review; the items/measures used in each study are listed in Table II. Finally, to accurately measure the effect size of the credibility-outcome relationship, the review was limited to studies that provided detailed statistical reporting, or where additional information was available after contacting the authors.

### Data Extraction and Coding

Articles included in this review were categorized into two groups based on their focus on credibility perception. Group 1 explored the relationship between perceived therapist credibility and treatment outcomes, while Group 2 examined the relationship between perceived treatment credibility and treatment outcomes. Data extraction was performed by the first author, following a standardized protocol, with credibility perception as the independent variable and post-treatment outcomes as the dependent variable. A second reviewer independently checked 25% of the data extraction regarding the effect sizes. Information collected included the publication year, country of data collection, sample size, participant age and gender, treatment modality, primary diagnosis, credibility and outcome measures, the timing of credibility assessment, study design, statistical analysis method, relevant findings, and effect sizes.

Gender characteristics were coded as all-female, all-male, or mixed samples, while diagnoses of the participants were given according to inclusion criteria in the relevant articles (see Table I, Table II). Treatment approaches were not coded, however, due to the fact that most of the studies combined participants from different treatment arms, precluding further investigations. The assessment of treatment credibility timing was coded in two different ways. Initially, it was classified into either “initial” (before treatment initiation) or “emergent” (after treatment initiation) categories, following the guidelines proposed by Hardy et al. (1995). This classification aimed to investigate the effect of treatment credibility without considering therapist-related or process-related variables. Subsequently, the assessment timing was coded as a continuous variable based on the session number in which credibility was assessed. This approach helped us explore whether the association between treatment credibility and outcomes shifted across treatment. In relation to therapist credibility, the papers that assessed credibility after the first session were coded as initial since a preliminary interaction with the therapist is necessary to assess the clinician’s credibility (Kasarabada et al., 2002). However, we were unable to code session-based perceived therapist credibility due to the limited information available in the eligible papers. Articles were also grouped by publication year, with three categories: 2000 and earlier, 2001–2011, and 2012–2023.

**Table I.** Overview of the therapist credibility studies.

Study	Sample *N* (female, male)	Credibility Measure (Timing)	Credibility Timing (Session)	Primary Outcome	Diagnosis	Effect Size (*r)* of Credibility Dimensions	Effect Size (*r*)
Bathje et al. (2022)	*Sample – 1 N* = 69	CRF- S	Emergent	CPC ^b^	None	Not calculated	.55
Bathje et al. (2022)	*Sample – 2 N* = 162	CRF- S	Emergent	CPC ^b^	None	Not calculated	.46
Farsimadan, et al. (2007)	*N* = 100; (53, 47)	CERS	Emergent (post-treatment)	GF^a^	None	Not calculated	.52
Gieselmann & Pietrowsky (2016)	*N* = 26; (13, 13)	Analog scale	Initial (1)	Procrastinate, DS ^a^	Procrastination	Att. = .40, Exp. = .43, Trust. = .03	.35
Grimes & Murdock (1989)	*N* = 29	CRF-S	Initial (1)	GF^a^	None	Att.= .38, Exp. = .43, Trust = .41	.43
Kasarabada et al. (2002)	*N* = 511 (286, 225)	EAC-B	Emergent (2)	Addiction ^a^	Addiction	Att. = 0, Exp. = 0, Trust. = 0	.01
LaCrosse (1980)	*N* = 36 (8, 28)	CRF	Both (1, post-treatment)	CPC ^a^	Addiction	Initial / Emergent Att. = .45/.51, Exp. = .56/.62, Trust. = .38/.47	.64
Lafferty, et al. (1989)	*N* = 60 (49, 11)	TCS	Emergent (post-treatment)	GF^a^	Outpatients	Not calculated	.35
Lawlor et al. (2017)	*N* = 109	STQ	Emergent (post-treatment)	DS, AS, Psychosis^a^	Psychosis	Not calculated	.26
Ramnerö & Öst (2007)	*N* = 59	TCRS	Emergent (4, 8, 12)	Agoraphobia ^b^	Agoraphobia	Support. = .06, Exp. = -.10, Trust. = -.08	-.02

*Note.* ^a^ individual outcome variables, ^b^ combined outcome variables. CRF, Counselor Rating Form Short; CERS, Counselor Effectiveness Rating Scale; EAC-B, Expectations About Counseling Scale-Brief Form; TCS, Therapist Credibility Scale; STQ, Satisfaction with

Therapy Questionnaire; TCRS, Therapist-Client Rating Scale; CPC, Client perceived change; GF, Global Functioning; DS, Depressive Symptoms

**Table II.** Overview of the treatment credibility studies.

Study	Sample *N* (female, male)	Credibility Measure (# of items)	Credibility Timing (Session)	Primary Outcome	Diagnosis	Effect Size (*r*)
Barlow, et al. (1992)	*N* = 40	CEQ (4)	Emergent (1)	AS^b^	GAD	.30
Barnicot et al. (2016)	*N *= 47	CEQ (4)	Initial (0)	SH^a^	BPD	.40
Borkovec & Costello (1993)*	*N* = 55 (36, 19)	CEQ (3)	Emergent (1)	AS^b^	GAD	0
Borkovec & Mathews (1988)*	*N* = 30 (17, 13)	CEQ (3)	Emergent (1)	AS^a^	GAD with PD	0
Borkovec, et al. (1987)	*N* = 30 (17, 13)	CEQ (4)	Emergent (1)	AS^a^	GAD	0
Borkovec, et al. (2002)*	*N* = 69 (45, 24)	CEQ (3)	Emergent (1)	AS^b^	GAD	.31
Carlbring et al. (2005)	*N* = 24 (18, 6)	CEQ (5)	Initial (0)	DS, AS^b^	PD	.25
Devilly & Spence (1999)	*N* = 22	CEQ (4)	Emergent (1)	PTSD, GF, DS^a^	PTSD	.18
Freeston et al. (1997)	*N* = 15	CEQ (6)	Emergent (2, post-treatment)	OCD, GF, DS, AS^a^	OCD	0
Greenberg et al. (2019)	*N* = 44 (27, 17)	CEQ (3)	Initial (0)	BDD^b^	BDD	.14
Hardy et al. (1995)	*Sample −1 N* = 59	CEQ (4)	Both (0, 1)	GF, DS^a^	GP	.42
Hardy et al. (1995)	*Sample −2 N* = 58	CEQ (4)	Both (0, 1)	GF, DS, IF^a^	GP	.002
Harrison et al. (2019)	*Sample −1 N* = 50	CEQ (3)	Initial (0)	DS ^a^	MDD	.18
Harrison et al. (2019)	*Sample – 2 N* = 43	CEQ (3)	Initial (0)	DS ^a^	MDD	.02
Hellström & Öst (1996)	*Sample-1 N* = 69	CEQ (4)	Initial (0)	AS, BS, FS^b^	SP	0
Hellström & Öst (1996)	*Sample-2 N* = 69	CEQ (4)	Initial (0)	AS, BS, FS^b^	SP	.32
Hundt et al. (2014)	*N* = 150 (75, 75)	CEQ (4)	Initial (0)	AS^a^	GAD	.17
Kim et al. (2015)	*N = *30	CEQ (4)	Emergent (1)	AS^a^	Anxiety	.50
Kuzminskaite et al. (2021)	*N *= 182 (116, 66)	EQ-Credibility (2)	Initial (0)	DS, GF^a^	MDD	0
Mooney et al. (2014)*	*N = *117 (69, 47)	CEQ (3)	Emergent (2)	DS, AS^b^	MDD, PD or GAD	.24
Morrison & Shapiro (1987)	*N* = 40 (17, 23)	CEQ (4)	Emergent (2)	GF, DS^b^	GP	.21
Phillips et al. (2021)	*N *= 109	CEQ (3)	Initial (0)	BDD^a^	BDD	.16
Ramnerö & Öst (2004)*	*N* = 73 (50, 23)	CEQ (4)	Emergent (1)	FS, AS^b^	PD with agoraphobia	-.03
Rosmarin et al. (2013)	*N* = 159 (98, 61)	CEQ (3)	Initial (0)	DS, GF, SH^a^	None	.06
Samantaray et al. (2023)	*N* = 116 (50, 66)	CEQ (3)	Both (0, 3)	OCD, AS^a^	OCD	.45
Taylor, (2003)	*N* = 45	CEQ	Emergent (2)	PTSD, AS, DS^a^	PTSD	0
Thompson-Hollands et al. (2014)*	*N* = 27	CEQ (3)	Emergent (2)	AS, DS, GF^a^	Anxiety	.03
Thornett & Mynors-Wallis (2002)	*N* = 148	CEQ (3)	Both (0,12)	DS^a^	MDD	0
Vos-Vromans et al. (2016)	*N* = 113 (92, 21)	CEQ (3)	Emergent (2)	Fatigue, GF, DS^a^	Chronic fatigue	0
Westra et al. (2011)*	*N* = 32 (23, 9)	CEQ (3)	Emergent (1, 3, 5, 7)	AS^a^	GAD	.45

*Note.* *Studies that were overlapped with previous meta-analysis. ^a^ individual outcome variables, ^b^ combined outcomes variables.

AS, anxiety symptoms; SH, self-harm; DS, depressive symptoms; GF, general functioning; IF, interpersonal functioning

GAD, general anxiety disorder; BPD, borderline personality disorder; PD, panic disorder; PTSD, post-traumatic stress disorder; OCD, obsessive compulsive disorder; BDD, body dysmorphic disorder; SP, specific phobia; MDD, major depressive disorder

Study quality was assessed by using the Effective Public Health Practice Project tool’s guidelines (Thomas et al., 2004), with interrater reliability between first and second authors reaching a substantial level (Cohen’s k = .70, *p* < .001). Results and dimensions of the quality assessment are presented in Supplementary Materials (see Table ST1). The certainty of the evidence for each group was evaluated independently by two authors using the Grading of Recommendations Assessment, Development, and Evaluation (GRADE) approach based on five domains: risk of bias, inconsistency, indirectness, imprecision, and publication bias (Schünemann et al., 2013). The determination of the certainty of evidence (whether it is high, moderate, low, or very low) was based on a consensus reached between the raters.

### Statistical Analysis Plan

Effect sizes were calculated using Pearson’s correlation coefficient, *r*, and the effect sizes reported with other statistical measures were converted to *r* (Harrer et al., 2021). The direction of the effect size was coded as a positive coefficient, indicating that a higher perception of credibility was associated with better outcomes at the end of treatment. However, some studies in the treatment credibility group only reported the credibility-outcome associations as nonsignificant, and they did not provide specific statistical values for the effect sizes. Where authors did not reply to requests for this data, a correlation of 0 was imputed for these studies (n = 9), following the procedure adopted by Constantino et al. (2018) which assumed a chance distribution of insignificant correlations around zero. Later, we repeated the analysis by excluding those studies and reported both analyses to avoid overestimation as suggested by Rosenthal (1995). Moreover, when contacting the authors of a study in the therapist credibility group for additional information (Bathje et al., 2022), it was found that a small fraction of participants (<10%) who underwent either group therapy or couples therapy were also included in the sample. Whilst we included this study in the primary analyses, a sensitivity analysis without this study is also reported.

To compute the credibility-outcome association in both groups separately, we initially conducted a two-level meta-analysis (unconditional or baseline model) by aggerating effect sizes from each study, assuming a correlation of *r *= .05 (Schmidt & Hunter, 2016). This initial step allowed us to perform diagnostic tests on the data and visualize the results in forest plots. However, it is important to note that the three-level meta-analysis method is widely regarded as the most statistically robust approach for addressing the independence of effect sizes (Assink & Wibbelink, 2016). Therefore, a multilevel meta-analytic model was tested, and adopted if it demonstrated a better fit to data, in order to preserve all data from the included studies and to offer a comprehensive understanding of the data variability while also estimating the effects of the subgroups, as suggested by Harrer et al. (2021). According to the multilevel model, Level 1 represented the sampling variance of the observed effect sizes (i.e., pooling the effect size of participants); Level 2 contained the variance from different effect sizes that were extracted from the same study (e.g., pooling the effect size of different outcomes within the same study); and, Level 3 captured variance between studies (i.e., pooling the overall true effect) (Assink & Wibbelink, 2016; Harrer et al., 2021).

All analyses were conducted using R statistical software (RStudio – 2022.07.2), including meta-analysis packages “Mac” (Del Re & Hoyt, 2012), “metafor” (Viechtbauer, 2010), and “meta” (Balduzzi et al., 2019). Correlation coefficients were converted to Fisher’s z to reduce potential bias from varying sample sizes, then the overall effect sizes were translated back to the correlation coefficient, r, for interpretation (Harrer et al., 2021). The effect sizes were interpreted based on established guidelines (Hemphill, 2003), whereby effect sizes below .20 were considered small, effect sizes equal to or greater than .20 were categorized as medium, and effect sizes equal to or greater than .30 were classified as large.

Because of the varied conceptualizations of treatment outcome and credibility, a random-effects model was used for all analyses. *Q* and *I*^2^ statistics were reported to assess within and between-study heterogeneity. We interpreted *I*^2^ values of 25% or less as negligible heterogeneity, values below 50% indicated moderate heterogeneity, and values exceeding 75% were taken to indicate high heterogeneity (Higgins et al., 2003). The restricted maximum-likelihood estimator (REML) was selected as the heterogeneity variance estimator for all meta-analyses, as recommended for continuous outcome meta-analyses of both small and large study sizes (Langan et al., 2019; Novianti et al., 2014). Additionally, outlier analyses, leave-one-out influence analyses (Viechtbauer & Cheung, 2010), and Baujat plots (Baujat et al., 2002) were conducted to detect studies that contributed to the between-study heterogeneity. Egger’s regression tests (Egger et al., 1997) were utilized to assess publication bias. Moreover, to explore heterogeneity, a set of meta-regression analyses was conducted to scrutinize potential moderators, which were chosen based on earlier research and data availability. These included the timing of the credibility assessment, the quality of the paper, and the year of publication. Other potential moderators such as participant gender, gender/ethnicity alignment with the therapist, primary diagnosis, and treatment modality were not considered due to either insufficient data or the amalgamation of different categories during the relevant analyses in the reviewed papers. The data and analysis codes that were used in the analyses are provided in a public repository (https://osf.io/nqzxs).

## Results

**Figure 1.**
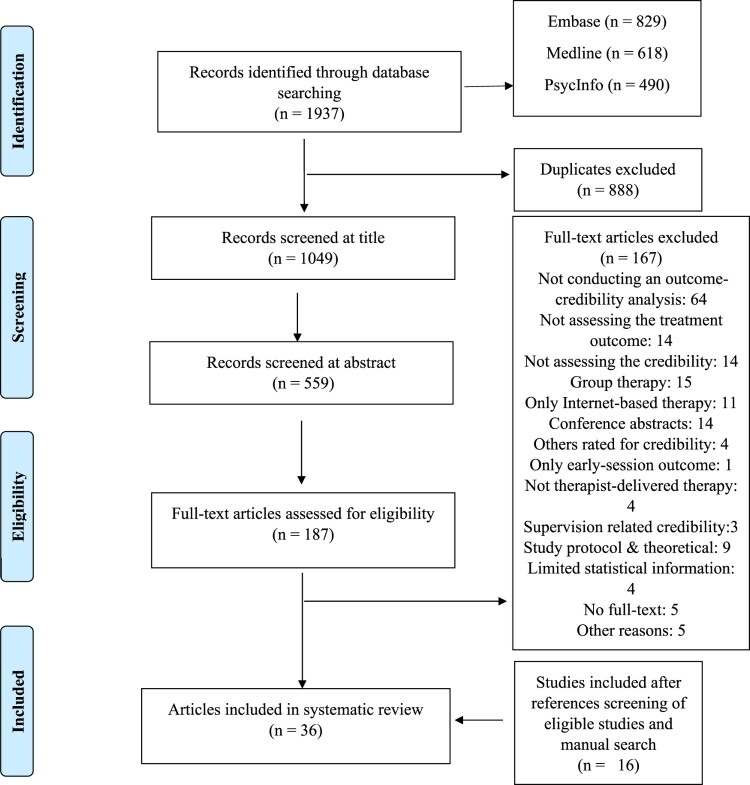
A flow diagram of the study selection.

The study selection process is depicted in Figure 1. Interrater reliability in the study selection process was high, with two authors reaching almost perfect agreement on abstract/title screening (Cohen’s *k* = .81, *p* < .001) and perfect agreement on full-text screening (Cohen’s *k *= 1, *p* < .001). Seven of the studies retrieved in the treatment credibility group overlapped with Constantino et al.’s (2018) previous meta-analysis and 20 new studies were identified (see Table II for details on the overlapping studies).

### Study Characteristics

Eligible papers were published between 1980 and 2023. For the therapist credibility group, 33% (n = 3) were published in the last decade (2013-2023), 33% (n = 3) between 2002 and 2012, and 33% (n = 3) before 2002. Regarding the treatment credibility, 48% (n = 13) were published in the last decade, 19% (n = 5) between 2002 and 2012, and 33% (n = 9) before 2002. The therapist credibility groups had a total sample size of 1161, while the treatment credibility group had 2061 participants. Therapist credibility group ages ranged from 15 to 68, with one study including 15–32-year-olds (adolescents and young adults), and treatment credibility group ages ranged from 18 to 90, including a study with participants over 60 years old. All studies involved mixed-gender samples.

A range of treatment modalities was observed, with cognitive behavioral therapy (CBT) being the most frequently used (n = 15), followed by cognitive therapy (n = 5) and counseling (n = 5). Other therapeutic approaches included psychodynamic therapy, interpersonal therapy, EMDR, dialectic behavioral therapy, exposure and systematic desensitization therapies, non-directive therapy, supportive therapy, problem-solving therapy, behavioral therapy, breathing therapy, and muscle relaxation. Table I, Table II present the primary diagnoses of the participants.

Measures of outcome and credibility varied across studies. In terms of outcome, some studies examined each outcome measure independently, while others combined multiple outcomes into a single variable, such as “treatment responder,” and focused on the composite scores of the therapy outcome (see Table I, Table II). Regarding credibility assessment timing, six studies in the treatment credibility group evaluated credibility multiple times throughout the therapy. Out of 30 independent samples, 50% (n = 15) assessed credibility before treatment began, 30% (n = 9) after session 1, 20% (n = 6) after session 2, and 13% (n = 4) after subsequent sessions. The session number in which credibility was measured for each study is detailed in Table II. In the therapist credibility group, two studies assessed credibility multiple times, while one study (11%) did not specify session number. Out of 10 independent samples, 33% (n = 3) of studies evaluated credibility after session 1, 10% (n = 1) after session 2, and 50% (n = 5) after subsequent sessions (see Table I).

The quality of the evidence in each meta-analysis was assessed using GRADE ratings. There were some concerns about limitations in study design, imprecision and publication bias when looking at the therapist credibility group. This was partly due to the limited number of included studies and the small number of participants in the existing studies. As a result, the quality of the evidence was downgraded to a “low” level. Similarly, the treatment credibility was downgraded to moderate quality of evidence because of problems with study limitations and suspected publication bias.

### The Relationship Between Therapist Credibility and Treatment Outcome

#### Unconditional model

A two-level meta-analysis with 10 independent samples and 1,161 participants was performed by aggregating all within-study effects (See Table I for pooled effect sizes for each study). The overall association between therapist credibility and treatment outcome indicated a large effect (*r* = .35, 95% CI = [.19, .50], *t* = 4.73. *p* = .001). Overall heterogeneity of the model was significant (*Q* (9) = 71.02, *p* < .001, *τ*^2 ^= .05, 95% CI = [.02, .17]) and the observed variability was high (*I*^2^ = 87.3%, 95% CI = [78.7%, 92.5%]). A forest plot of the two-level model with study weights is shown in the Supplementary Materials (see Figure SF1).

After outlier analysis and *leave-one-out* sensitivity analyses were performed and influence diagnostics visually examined, we observed that one study (Kasarabada et al., 2002) influenced heterogeneity greatly. This could be attributed to the notably larger sample size in this study compared to others within the same group. Analysis was repeated after eliminating this study, and the overall effect remained significant with an increase in correlation coefficient (*r* = .40, 95% CI = [.25, .52], *t* = 5.97. *p* < .001). Removing this study also decreased heterogeneity to a moderate level (*I*^2^ = 62.7%, 95% CI = [23.3%, 81.9%]), although it remained significant (*Q* (8) = 21.46, *p* = .006, *τ*^2 ^= .03, 95% CI = [.003, .14]). The findings of the Egger's test did not provide evidence to support the presence of funnel plot asymmetry (*t* = -.21, *p* = .84).

#### Three-level model

To evaluate the adequacy and capacity of a three-level model to better capture data variability than the two-level model, the goodness of fit of these models was compared. Results revealed that the complexity of the three-level model was justified with a significantly better fit to the data than the two-level model (X^2^_1_ = 61.38, *p *< .001). For the multilevel meta-analysis with 44 effect sizes, the overall effect of therapist credibility on treatment outcome was statistically significant with a large effect (*r* = .35, 95% CI = [.18, .51], *t *= 4.18, *p* < .001, GRADE = low). However, overall heterogeneity was statistically significant (Q (41) = 132.58, *p* < .001). The estimated variance components were τ^2^_Level 3 _= 0.06 and τ^2^_Level 2 _=  0, and *I*^2^_Level 3 _= 88.99% of the total variation was attributed to between-study heterogeneity.

Finally, sensitivity analyses were conducted without Bathje et al. (2022). Similar to previous results, the overall association between therapist credibility and treatment outcome was significant for both two-level meta-analysis (*r* = .29, 95% CI = [.10, .48], *t* = 3.53, *p* = .01) and multilevel analyses (*r* = .30, 95% CI = [.11, .47], *t* = 3.21, *p* < .001). Levels of heterogeneity remained statistically significant (*Q* (39) = 95.69, *p* < .001, *I*^2^ = 89.2%).

#### Moderators of the relationship between therapist credibility and treatment outcome

The substantial heterogeneity observed across studies on therapist credibility necessitated conducting moderator analyses to investigate the influence of potential factors on the relationship between therapist credibility and treatment outcome. The findings suggest that the association between credibility and outcome did not exhibit a statistical difference (*F*(1, 40) = .38, *p* = .54) when participants reported their perceptions of credibility after session 1 (*r* = .42, *p* = .01) as compared to emergent credibility perceptions (*r *= .32, *p* = .54). Similarly, neither the quality of the studies (*F*(2, 39) =  1.22, *p* = .31) nor the publication year (*F*(2, 39) =  1.48, *p* = .24) emerged as significant moderators.

#### Individual aspects of therapist credibility

For additional information, we explored the effect of individual aspects of therapist credibility on the treatment outcome and described our initial results in a narrative synthesis owing to the small number of studies. Out of five studies that reported therapist credibility aspects separately, two studies revealed that perceived therapist expertness, attractiveness, and trustworthiness predicted the treatment outcome while another study revealed both attractiveness and competence were associated with a better outcome. The most powerful factor associated with effect size across studies was perceived expertness (see Table I for the effect sizes).

### The Relationship Between Treatment Credibility and Treatment Outcome

#### Unconditional model

A two-level meta-analysis was conducted with 30 independent samples and a total of 2,061 participants (See Table II for aggregated effect sizes). The overall treatment credibility – outcome correlation was calculated by pooling all within study effects and results revealed a small but significant association (*r* = .16, 95% CI = [.09, .22], *t* = 4.80, *p* < .001). Overall heterogeneity was significant (*Q* (29) = 57.43, *p* < .001, *τ*^2 ^= .02, 95% CI = [.003, .04]) and the observed variability between studies was small to moderate (*I*^2^ = 49.5%, 95% CI = [22.9%, 66.9%]). A forest plot of the aggregated effect sizes is presented in the Supplementary Materials (see Figure SF2).

The Egger’s test was not statistically significant, indicating there was no evidence for publication bias (*t* = 1.14, *p* = .26). The outlier analysis and influence diagnostics identified one outlier (Samantaray et al., 2023). This could be due to the naturalistic mental health setting of the study, which resulted in a lower quality assessment score and increased susceptibility to various confounders. Analyses were repeated without this outlier, and the overall effect remained significant with a minimal decrease in effect size (*r* = .14, 95% CI = [.07, .20], *t *= 4.47, *p* < .001). Furthermore, removing this study from the analysis decreased the heterogeneity to a negligible level (*I*^2^ = 35.5%, 95% CI = [0%, 58.9%]), although it remained significant (*Q* (28) = 43.41, *p* = .03, *τ*^2 ^= .01, 95% CI = [.00, .03]).

#### Three-level model

The three-level model demonstrated a better fit to data and an increased ability to explain data variability compared to the simpler two-level model (X^2^_1_ = 23.10, *p* < .001). Results of the pooled correlation based on the three-level model with 70 effect sizes revealed a small but significant association (*r* = .15, 95% CI = [.09, .21], *t *= 4.85, *p* < .001). Findings indicated that higher treatment credibility perception was associated with better treatment outcomes (GRADE = moderate). A small to moderate level heterogeneity was observed (*Q* (69) = 113.54, *p* < .001, *I*^2^ = 42.46%). The estimated variance components were *τ*^2^_Level 3 _= .01 and *τ*^2^_Level 2 _=  .00. Heterogeneity analyses revealed that *I*^2^_Level 3 _= 42.46% of the total variation can be attributed to between-study heterogeneity and less than 1% to sampling error and within-study heterogeneity.

#### Moderators of the relationship between treatment credibility and treatment outcome

Consistent with previous findings, none of the examined moderators including the quality of the studies (*F*(2, 67) = .94, *p* = .39) and the publication year (*F*(2, 67) =  .63, *p* = .54) reached statistical significance. The results indicated that there was no statistically significant distinction in the correlation between participants’ initial reporting of credibility perceptions (*r* = .14, *p* < .001) and their emergent perceptions (*r* = .15, *p* = .79) in relation to outcome (*F*(1, 68) = .07, *p* = .79). Finally, we explored the change in perceived treatment credibility across sessions, however, no significant difference was found (*F*(1, 67) = .08, *p* = .78).

#### Analyses without missing value imputation for non-significant articles

Finally, analyses were repeated excluding studies that reported their results as nonsignificant without giving detailed statistical information, rather than imputing 0 as their effect sizes (see Supplementary Material 2 for detailed results). Similar to previous results, the mean of the effect sizes regarding treatment credibility - outcome relationship indicated a small association (*r* = .19, 95% CI = [.13, .27], *t* = 5.57, *p* < .001). A moderate level of heterogeneity was observed (*Q* (58) = 94.72, *p* = .002, *I*^2^ = 42.34). The results of the moderator analyses also remained the same.

## Discussion

The findings of this review indicate a strong positive association between perceived therapist credibility and therapeutic change, as well as a smaller effect between treatment credibility and change. The robustness of the findings was assessed in a three-level meta-analysis; however, none of the available moderators was associated with the observed effect size. There were fewer studies that included measures of therapist credibility, and high levels of heterogeneity were observed indicating the potential for further work to better understand this relationship.

However, the strong association between perceived therapist credibility and treatment outcomes potentially supports the contextual model, which states the client’s evaluation of the therapist's trustworthiness and expertise is an important factor in contributing to therapeutic change (Wampold, 2015). Moreover, our findings suggest that perceptions of therapist credibility, although often overlooked compared to treatment credibility, could be important when comparing and evaluating the effectiveness of therapies. When evaluating the therapist's credibility in more detail, we found that the client's perception of the therapist's expertise was more strongly associated with better therapeutic results than other aspects including trustworthiness. This emphasizes credibility as a broad concept, which is not only limited to interpersonal trust or trustworthiness, but also takes into account the perception of competence.

Our findings revealed no marked difference between clients’ initial and subsequent perceptions of therapist credibility in relation to their post-treatment outcomes. This underscores the significance of therapist credibility even before any tangible progress is observed or a therapeutic bond is established. While these results help distinguish from potential confounding variables, they also prompt deeper reflection on client attributes that might shape credibility perceptions. We hypothesize that while credibility is frequently associated with the perceived attributes of the therapist, it might also be anchored in the client's innate capacity to trust, a facet tied to their personality characteristics (Caesar, 1996). Previous theoretical papers have asserted the importance of basic trust in the therapeutic relationship (Laughton-Brown, 2010); however, the findings of this review emphasize the role of a particular aspect of trust between the therapist and client that is closely related to knowledge. This aspect mainly depends on the thought processes in which the client evaluates the expertise of the therapist. Fonagy and Allison (2014) describe this aspect as epistemic trust, which refers to the capacity to receive and perceive interpersonally conveyed knowledge as relevant to self, reliable and generalizable to other situations. Disruptions in epistemic trust, as Allison and Fonagy (2016) suggest, can result in perceiving received information as non-relevant or untruthful, which in turn can impede the client's ability to internalize new information and benefit from psychotherapy.

The results of our meta-analysis on treatment credibility align with the findings of Constantino et al. (2018), showing a slightly stronger relationship between treatment credibility and outcome. Although seven studies from our review also appeared in the previous one, differences in inclusion criteria led to variations in the studies selected for each review. The earlier review included diverse treatment modalities, such as group therapy and online therapy. However, we intentionally excluded these modalities in our study due to potential variations in credibility perception within them and their potential to confound the results (Söchting et al., 2016). Moreover, the current meta-analysis extends that of previous research by considering a more detailed analysis of the effects of the credibility perceptions and employing a three-level model that evaluated both within-study and between-study variability. Results revealed a small degree of heterogeneity between outcomes within the same sample, which suggests that the direct effect of treatment credibility on specific outcomes might be negligible. Instead, its primary influence on outcome may be through its impact on general well-being (Thompson-Hollands et al., 2014) This finding is also in line with past research which has demonstrated that treatment credibility measurement is commonly employed to control for the placebo effect (Borkovec & Nau, 1972) and is associated with general therapeutic improvement (Devilly & Borkovec, 2000).

Building upon the previous meta-analysis, our study incorporated credibility evaluations taken at different treatment phases. By including pre-treatment credibility assessments, we could uniquely explore the influence of treatment credibility separately from therapist-related factors, such as therapist credibility or the congruence between therapist and client (Hardy et al., 1995). It is suggested that once therapy commences, various elements, including therapy engagement, early symptom alleviation, and the therapeutic alliance, can mold credibility beliefs (Westra et al., 2011). A pivotal discovery from our research is the link between clients’ pre-treatment credibility views and their post-treatment psychological well-being. Notably, this relationship mirrored the correlation observed between post-treatment psychological health and credibility perceptions formed after therapy began. Mooney et al. (2014) previously suggested that positive treatment perceptions stem from early treatment experiences rather than initial characteristics, influencing eventual outcomes. Contrarily, our findings emphasize the significance of pinpointing initial client traits that might sway credibility perceptions, as indicated by Constantino et al. (2014). Factors such as a client's prior treatment experiences, initial well-being, preconceived beliefs about therapy effectiveness, and their capacity for epistemic trust could potentially shape their credibility perceptions.

Only a handful of studies have assessed perceptions of treatment credibility in later stages. Prior research posits that credibility is a fluid construct, evolving throughout therapy, rather than a fixed belief (Constantino et al., 2018). Our data indicates a consistent significant relationship between treatment credibility and its effectiveness throughout the therapeutic journey. This insight is especially relevant for studies probing treatment credibility early on, especially concerning the placebo effect. It might be beneficial to gauge the variances in perceived treatment credibility not just at the onset but also during advanced treatment phases. However, it is worth noting that the majority of studies we analyzed overlooked factors such as early symptom relief or the therapeutic alliance, both of which could shape subsequent credibility perceptions. Furthermore, this relationship might be reciprocal; the impact of treatment credibility on results may be indirect, channeled through other prevalent factors influencing outcomes. For instance, the working alliance has been identified as a strong predictor of symptom improvement after treatment (Flückiger et al., 2018), and higher treatment credibility has been found to be a significant predictor of the working alliance (Fjermestad et al., 2018).

In summary, our findings are consistent with the Common Factors Theory, suggesting that various treatment modalities may produce similar outcomes due to underlying shared mechanisms (Imel & Wampold, 2008). Our results provide evidence that the client's perception of credibility, either concerning the treatment or therapist, is a significant contributing factor to therapeutic efficacy.

### Limitations

The certainty of evidence as assessed by the GRADE approach was low for therapist credibility and moderate for the treatment credibility group. The limitations of the evidence arise from both the limitations in the included studies and the review. Firstly, the number of eligible studies and sample sizes within the analyses were small, particularly in the therapist credibility group, and this alongside the poorer quality of some studies may limit the generalisability of findings. A number of potentially important confounders in the relationship between credibility and outcome such as early symptom improvement, and ethnic/gender match between the therapist and the client were not considered in studies. Additionally, some articles included behavior tests or one-item measures to evaluate outcomes that lacked reliability and validity reports, and the variability in items to measure credibility are further limitations. This variability may lead to mixed results, as it could mean that some studies blended credibility items with expectancy items, or used these constructs interchangeably (Devilly & Borkovec, 2000). Furthermore, some papers analysed the relationship between credibility and treatment outcome separately for each treatment arm, and others combined all participants. Additionally, many of the qualifying articles were identified through manual searches and references, as the majority of studies did not mention credibility in their abstracts. Another constraint was our language-based inclusion criteria, which omitted non-English articles. This exclusion might render our results vulnerable to publication bias and limit their applicability to a broader audience. Lastly, the primary author largely undertook the screening and data extraction from the selected studies. Even though other reviewers independently assessed a portion of the studies at various stages, this approach might introduce potential biases.

### Future Studies and Clinical Implications

The current review identified the significant roles of perceptions of therapists and treatment credibility on the treatment outcomes and suggested areas for further discussion and research. Our findings emerged from a sample of varied-quality papers. Future studies should seek to replicate these findings using more representative and larger samples, as well as employing structured assessments for both credibility and outcome and controlling for other possible confounding variables (e.g., ethnic/gender match of the therapist and the client, type and duration of treatment). Furthermore, our review only indicates a correlation between credibility perceptions and the treatment outcome, thus future studies that experimentally manipulate credibility perceptions are warranted to explore this relationship in a causal framework. Moreover, the roles of other common factors such as working alliance in the relationship between credibility and outcome should be further investigated alongside the credibility of both therapy and therapist. Additionally, one of our arguments was that the client’s credibility belief may be linked to an individual’s capability of epistemic trust. Future studies should work to explore this assumption.

This review concludes by reflecting on the clinical ramifications of our findings. We discerned a link between clients’ heightened credibility perceptions and superior therapeutic outcomes. While various influencing factors cannot be disregarded, within the prevailing theoretical context, we posit that addressing credibility perceptions can be advantageous. A strategy to enhance this might be by devising a treatment rationale explanation tailored to the client's unique needs and viewpoints, factoring in the client’s existing beliefs about the treatment, challenges, and their self-concept (Coyne et al., 2019). Clinicians might also find value in gauging a client's views on the credibility of the suggested treatment through concise structured tools or by seeking feedback on their sentiments and apprehensions about the therapy. While multiple facets of the therapeutic bond might sway credibility and outcomes in therapy's advanced stages, our data indicates that credibility's influence remains steadfast. Therapists should thus remain vigilant to instances where clients seem skeptical of the therapy or therapist, not just initially but throughout the therapeutic journey. Open dialogues addressing any perceived inconsistencies, irrelevancies, or illogicalities can be fruitful. If certain therapeutic methods seem unconvincing, therapists should elucidate their rationale and tweak the approach as needed. In advanced stages of therapy, the focus should encompass both the relational and logical aspects of treatment. Our insights into pre-treatment credibility perceptions underscore the therapist's role in discerning pivotal traits influencing credibility beliefs. By pinpointing clients with credibility vulnerabilities, therapists can preemptively address their doubts during initial sessions, amplifying the therapy's potential benefits. Lastly, psychotherapists can be trained to track clients’ beliefs and adapt their conduct to bolster credibility perceptions. Tactics like sustained eye contact, adopting a “forward trunk lean” posture (Dowell & Berman, 2013), and presenting treatment details in a comprehensible manner are methods clinicians can utilize to enhance clients’ credibility views (Horvath, 1990).

## Supplementary Material

tpsr-2023-0236-File005

## References

[CIT0001] Allison, E., & Fonagy, P. (2016). When is truth relevant? *The Psychoanalytic Quarterly*, *85*(2), 10.1002/psaq.12074 27112740

[CIT0002] Apolinário-Hagen, J., Harrer, M., Kählke, F., Fritsche, L., Salewski, C., & Ebert, D. D. (2018). Public attitudes toward guided internet-based therapies: Web-based survey study. *JMIR Mental Health*, *5*(2), 10.2196/10735 PMC5974457 29764797

[CIT0003] Assink, M., & Wibbelink, C. J. M. (2016). Fitting three-level meta-analytic models in R: A step-by-step tutorial. *The Quantitative Methods for Psychology*, *12*(3), 10.20982/tqmp.12.3.p154

[CIT0004] Balduzzi, S., Rücker, G., & Schwarzer, G. (2019). How to perform a meta-analysis with R: A practical tutorial. *Evidence Based Mental Health*, *22*(4), 10.1136/ebmental-2019-300117 PMC10231495 31563865

[CIT0005] Barlow, D. H., Rapee, R. M., & Brown, T. A. (1992). Behavioral treatment of generalized anxiety disorder. *Behavior Therapy*, *23*(*4*), 551–570. 10.1016/S0005-7894(05)80221-7

[CIT0006] Barnicot, K., Gonzalez, R., McCabe, R., & Priebe, S. (2016). Skills use and common treatment processes in dialectical behaviour therapy for borderline personality disorder. *Journal of Behavior Therapy and Experimental Psychiatry*, *52*, 147–156. 10.1016/j.jbtep.2016.04.006 27132175

[CIT0007] Bathje, G. J., Pillersdorf, D., & Eddir, H. (2022). Multicultural competence as a common factor in the process and outcome of counseling. *Journal of Humanistic Psychology*, 10.1177/00221678221099679

[CIT0008] Baujat, B., Mahé, C., Pignon, J. P., & Hill, C. (2002). A graphical method for exploring heterogeneity in meta-analyses: Application to a meta-analysis of 65 trials. *Statistics in Medicine*, *21*(18), 10.1002/sim.1221 12228882

[CIT0009] Bordin, E. S. (1979). The generalizability of the psychoanalytic concept of the working alliance. *Psychotherapy: Theory, Research & Practice*, *16*(3), 10.1037/h0085885

[CIT0010] Borkovec, T. D., & Costello, E. (1993). Efficacy of applied relaxation and cognitive-behavioral therapy in the treatment of generalized anxiety disorder. *Journal of Consulting and Clinical Psychology*, *61*(*4*), 611–619. 10.1037/0022-006X.61.4.611 8370856

[CIT0011] Borkovec, T. D., & Mathews, A. M. (1988). Treatment of nonphobic anxiety disorders: A comparison of nondirective, cognitive, and coping desensitization therapy. *Journal of Consulting and Clinical Psychology*, *56*(*6*), 877–884. 10.1037/0022-006X.56.6.877 2904928

[CIT0012] Borkovec, T. D., Mathews, A. M., Chambers, A., Ebrahimi, S., Lytle, R., & Nelson, R. (1987). The effects of relaxation training with cognitive or nondirective therapy and the role of relaxation-induced anxiety in the treatment of generalized anxiety. *Journal of Consulting and Clinical Psychology*, *55*(*6*), 883–888. 10.1037/0022-006X.55.6.883 3320121

[CIT0013] Borkovec, T. D., & Nau, S. D. (1972). Credibility of analogue therapy rationales. *Journal of Behavior Therapy and Experimental Psychiatry*, *3*(4), 257–260. 10.1016/0005-7916(72)90045-6

[CIT0014] Borkovec, T. D, Newman, M. G., Pincus, A. L., & Lytle, R. (2002). A component analysis of cognitive-behavioral therapy for generalized anxiety disorder and the role of interpersonal problems. *Journal of Consulting and Clinical Psychology*, *70*(*2*), 288–298. 10.1037/0022-006X.70.2.288 11952187

[CIT0015] Caesar, C. A. (1996). *Clients’ perceived trust, trustworthiness, and expectations in the therapeutic relationship: Factors in satisfaction with counseling*. University of Florida.

[CIT0016] Carlbring, P., Nilsson-Ihrfelt, E., Waara, J., Kollenstam, C., Buhrman, M., Kaldo, V., Söderberg, M., Ekselius, L., & Andersson, G. (2005). Treatment of panic disorder: live therapy vs. self-help via the Internet. *Behaviour Research and Therapy*, *43*(*10*), 1321–1333. 10.1016/j.brat.2004.10.002 16086983

[CIT0017] Constantino, M. J., Coyne, A. E., Boswell, J. F., Iles, B. R., & Visla, A. (2018). A meta-analysis of the association between patients’ early perception of treatment credibility and their posttreatment outcomes. *Psychotherapy*, *55*(4), 486–495. 10.1037/pst0000168 30335460

[CIT0018] Constantino, M. J., Penek, S., Bernecker, S. L., & Overtree, C. E. (2014). A preliminary examination of participant characteristics in relation to patients’ treatment beliefs in psychotherapy in a training clinic. *Journal of Psychotherapy Integration*, *24*(3), 10.1037/a0031424

[CIT0019] Coste, J., Tarquinio, C., Rouquette, A., Montel, S., & Pouchot, J. (2020). Cross-cultural adaptation and validation of the French version of the credibility/expectancy questionnaire. Further insights into the measured concepts and their relationships. *Psychologie Française*, *65*(2), 173–184. 10.1016/j.psfr.2018.11.001

[CIT0020] Coyne, A. E., Constantino, M. J., & Muir, H. J. (2019). Therapist responsivity to patients’ early treatment beliefs and psychotherapy process. *Psychotherapy*, *56*(1), 10.1037/pst0000200 30688483

[CIT0021] Cuijpers, P., Reijnders, M., & Huibers, M. J. H. (2019). The role of common factors in psychotherapy outcomes. *Annual Review of Clinical Psychology*, *15*(1), 10.1146/annurev-clinpsy-050718-095424 30550721

[CIT0022] Del Re, A. C., & Hoyt, W. T. (2012). *MAc: Meta-analysis with correlations* (R package version 1.1). https://cran.r-project.org/src/contrib/Archive/MAc/.

[CIT0023] Devilly, G. J., & Borkovec, T. D. (2000). Psychometric properties of the credibility/expectancy questionnaire. *Journal of Behavior Therapy and Experimental Psychiatry*, *31*(2), 10.1016/S0005-7916(00)00012-4 11132119

[CIT0024] Devilly, Grant J., & Spence, Susan H. (1999). The relative efficacy and treatment distress of EMDR and a cognitive-behavior trauma treatment protocol in the Amelioration of posttraumatic stress disorder. *Journal of Anxiety Disorders*, *13*(*1-2*), 131–157. 10.1016/S0887-6185(98)00044-9 10225505

[CIT0025] Dowell, N. M., & Berman, J. S. (2013). Therapist nonverbal behavior and perceptions of empathy, alliance, and treatment credibility. *Journal of Psychotherapy Integration*, *23*(2), 10.1037/a0031421

[CIT0026] Dryden, W., & Sabelus, S. (2012). The perceived credibility of two rational emotive behavior therapy rationales for the treatment of academic procrastination. *Journal of Rational-Emotive & Cognitive-Behavior Therapy*, *30*(1), 10.1007/s10942-010-0123-z

[CIT0027] Egger, M., Smith, G. D., Schneider, M., & Minder, C. (1997). Bias in meta-analysis detected by a simple, graphical test. *BMJ*, *315*(7109), 10.1136/bmj.315.7109.629 PMC2127453 9310563

[CIT0028] Farsimadan, F., Draghi-Lorenz, R., & Ellis, J. (2007). Process and outcome of therapy in ethnically similar and dissimilar therapeutic dyads. *Psychotherapy Research*, *17*(*5*), 567–575. 10.1080/10503300601139996

[CIT0029] Fjermestad, K. W., Lerner, M. D., McLeod, B. D., Wergeland, G. J. H., Haugland, B. S. M., Havik, O. E., Öst, L. G., & Silverman, W. K. (2018). Motivation and treatment credibility predict alliance in cognitive behavioral treatment for youth with anxiety disorders in community clinics. *Journal of Clinical Psychology*, *74*(6), 10.1002/jclp.22551 29143977

[CIT0030] Flückiger, C., Del, A. C., Wampold, B. E., & Horvath, A. O. (2018). The alliance in adult psychotherapy: A meta-analytic synthesis. *Psychotherapy*, *55*(4), 10.1037/pst0000172 29792475

[CIT0031] Fonagy, P., & Allison, E. (2014). The role of mentalizing and epistemic trust in the therapeutic relationship. *Psychotherapy*, *51*(3), 372–380. 10.1037/a0036505 24773092

[CIT0032] Freeston, M. H., Ladouceur, R., Gagnon, F., Thibodeau, N., Rhéaume, J., Letarte, H., & Bujold, A. (1997). Cognitive—behavioral treatment of obsessive thoughts: A controlled study. *Journal of Consulting and Clinical Psychology*, *65*(*3*), 405–413. 10.1037/0022-006X.65.3.405 9170763

[CIT0033] Gieselmann, A., & Pietrowsky, R. (2016). Treating procrastination chat-based versus face-to-face: An RCT evaluating the role of self-disclosure and perceived counselor's characteristics. *Computers in Human Behavior*, *54*, 444–452. 10.1016/j.chb.2015.08.027

[CIT0034] Greenberg, J. L., Phillips, K. A., Steketee, G., Hoeppner, S. S., & Wilhelm, S. (2019). Predictors of response to cognitive-behavioral therapy for body dysmorphic disorder. *Behavior Therapy*, *50*(*4*), 839–849. 10.1016/j.beth.2018.12.008 31208692 PMC6582981

[CIT0035] Grimes, W. R., & Murdock, N. L. (1989). Social influence revisited: Effects of counselor influence on outcome variables. *Psychotherapy: Theory, Research, Practice, Training*, *26*(*4*), 469–474. 10.1037/h0085465

[CIT0036] Hardy, G. E., Barkham, M., Shapiro, D. A., Reynolds, S., Rees, A., & Stiles, W. B. (1995). Credibility and outcome of cognitive—behavioural and psychodynamic—interpersonal psychotherapy. *British Journal of Clinical Psychology*, *34*(4), 555–569. 10.1111/j.2044-8260.1995.tb01489.x 8563662

[CIT0037] Harrer, M., Cuijpers, P., Furukawa, T. A., & Ebert, D. D. (2021). *Doing meta-analysis with R: A hands-On guide*. Chapman & Hall/CRC Press.

[CIT0038] Harrison, P., Hardy, G. E., & Barkham, M. (2019). The relationship between expected engagement and talking therapy outcome. *Clinical Psychology & Psychotherapy*, *26*(4), 492–501. 10.1002/cpp.2369 31018017 PMC6772155

[CIT0039] Hatcher, R. L. (2021). Responsiveness, the relationship, and the working alliance in psychotherapy. In J. C. Watson & H. Wiseman (Eds.), *The responsive psychotherapist: Attuning to clients in the moment* (pp. 37–58). American Psychological Association. 10.1037/0000240-003.

[CIT0040] Hellström, K., & Öst, L.-G. (1996). Prediction of outcome in the treatment of specific phobia. A cross validation study. *Behaviour Research and Therapy*, *34*(*5-6*), 403–411. 10.1016/0005-7967(96)00004-6 8687362

[CIT0041] Hemphill, J. F. (2003). Interpreting the magnitudes of correlation coefficients. *American Psychologist*, *58*(1), 10.1037/0003-066X.58.1.78 12674822

[CIT0042] Higgins, J. P. T., Thompson, S. G., Deeks, J. J., & Altman, D. G. (2003). Measuring inconsistency in meta-analyses. *British Medical Journal*, *327*(7414), 10.1136/bmj.327.7414.557 PMC192859 12958120

[CIT0043] Horvath, A. O., & Symonds, B. D. (1991). Relation between working alliance and outcome in psychotherapy: A meta-analysis. *Journal of Counseling Psychology*, *38*(2), 139–149. 10.1037/0022-0167.38.2.139

[CIT0044] Horvath, P. (1990). Treatment expectancy as a function of the amount of information presented in therapeutic rationales. *Journal of Clinical Psychology*, *46*(5), 10.1002/1097-4679(199009)46:5<636::AID-JCLP2270460516>3.0.CO;2-U 2246373

[CIT0045] Hundt, N. E., Amspoker, A. B., Kraus-Schuman, C., Cully, J. A., Rhoades, H., Kunik, M. E., & Stanley, M. A. (2014). Predictors of CBT outcome in older adults with GAD. *Journal of Anxiety Disorders*, *28*(*8*), 845–850. 10.1016/j.janxdis.2014.09.012 25445074 PMC4254548

[CIT0046] Imel, Z. E., & Wampold, B. E. (2008). The importance of treatment and the science of common factors in psychotherapy. In D. Brown & R. W. Lent (Eds.), *Handbook of counseling psychology* (pp. 249–266). John Wiley & Sons, Inc.

[CIT0047] Jacobson, N. S., & Baucom, D. H. (1977). Design and assessment of nonspecific control groups in behavior modification research. *Behavior Therapy*, *8*(4), 10.1016/S0005-7894(77)80203-7

[CIT0048] Kasarabada, N. D., Hser, Y.-I., Boles, S. M., & Huang, Y. C. (2002). Do patients’ perceptions of their counselors influence outcomes of drug treatment? *Journal of Substance Abuse Treatment*, *23*(4), 327–334. 10.1016/S0740-5472(02)00276-3 12495794

[CIT0049] Kazdin, A. E. (1979). Therapy outcome questions requiring control of credibility and treatment-generated expectancies. *Behavior Therapy*, *10*(1), 81–93. 10.1016/S0005-7894(79)80011-8

[CIT0050] Kim, S., Roth, W. T., & Wollburg, E. (2015). Effects of therapeutic relationship, expectancy, and credibility in breathing therapies for anxiety. *Bulletin of the Menninger Clinic*, *79*(*2*), 116–130. 10.1521/bumc.2015.79.2.116 26035087

[CIT0051] Kuzminskaite, E., Lemmens, L. H. J. M, van Bronswijk, S. C., Peeters, F., & Huibers, M. J. H. (2021). Patient choice in depression psychotherapy: Outcomes of patient-preferred therapy versus randomly allocated therapy. *American Journal of Psychotherapy*, appi.apt.2020.2. 10.1176/appi.apt.2020.2020.0042 34029118

[CIT0052] LaCrosse, M. B. (1980). Perceived counselor social influence and counseling outcomes: Validity of the Counselor Rating Form. *Journal of Counseling Psychology*, *27*(*4*), 320–327. 10.1037/0022-0167.27.4.320

[CIT0053] Lafferty, P., Beutler, L. E., & Crago, M. (1989). Differences between more and less effective psychotherapists: A study of select therapist variables. *Journal of Consulting and Clinical Psychology*, *57*(*1*), 76–80. 10.1037//0022-006X.57.1.76 2925976

[CIT0054] Langan, D., Higgins, J. P. T., Jackson, D., Bowden, J., Veroniki, A. A., Kontopantelis, E., Viechtbauer, W., & Simmonds, M. (2019). A comparison of heterogeneity variance estimators in simulated random-effects meta-analyses. *Research Synthesis Methods*, *10*(1), 10.1002/jrsm.1316 30067315

[CIT0055] Laughton-Brown, H. (2010). Trust in the therapeutic relationship: Psychodynamic contributions to counselling psychology practice. *Counselling Psychology Review*, *25*(2).

[CIT0056] Lawlor, C., Sharma, B., Khondoker, M., Peters, E., Kuipers, E., & Johns, L. (2017). Service user satisfaction with cognitive behavioural therapy for psychosis: Associations with therapy outcomes and perceptions of the therapist. *British Journal of Clinical Psychology*, *56*(*1*), 84–102. 10.1111/bjc.2017.56.issue-1 27910997

[CIT0057] Mooney, T. K., Gibbons, M. B. C., Gallop, R., Mack, R. A., & Crits-Christoph, P. (2014). Psychotherapy credibility ratings: Patient predictors of credibility and the relation of credibility to therapy outcome. *Psychotherapy Research*, *24*(5), 565–577. 10.1080/10503307.2013.847988 24219179 PMC4560353

[CIT0058] Morrison, L. A., & Shapiro, D. A. (1987). Expectancy and outcome in prescriptive vs. exploratory psychotherapy. *British Journal of Clinical Psychology*, *26*(*1*), 59–60. 10.1111/bjc.1987.26.issue-1 3828598

[CIT0059] Newman, M. G., & Fisher, A. J. (2010). Expectancy/credibility change as a mediator of cognitive behavioral therapy for generalized anxiety disorder: Mechanism of action or proxy for symptom change? *International Journal of Cognitive Therapy*, *3*(3), 245–261. 10.1521/ijct.2010.3.3.245 21132075 PMC2995495

[CIT0060] Nock, M. K., Ferriter, C., & Holmberg, E. (2007). Parent beliefs about treatment credibility and effectiveness: Assessment and relation to subsequent treatment participation. *Journal of Child and Family Studies*, *16*(1), 10.1007/s10826-006-9064-7

[CIT0061] Novianti, P. W., Roes, K. C. B., & van der Tweel, I. (2014). Estimation of between-trial variance in sequential meta-analyses: A simulation study. *Contemporary Clinical Trials*, *37*(1), 10.1016/j.cct.2013.11.012 24321246

[CIT0062] Page, M. J., McKenzie, J. E., Bossuyt, P. M., Boutron, I., Hoffmann, T. C., Mulrow, C. D., Shamseer, L., Tetzlaff, J. M., Akl, E. A., Brennan, S. E., Chou, R., Glanville, J., Grimshaw, J. M., Hróbjartsson, A., Lalu, M. M., Li, T., Loder, E. W., Mayo-Wilson, E., McDonald, S., … Moher, D. (2021). The PRISMA 2020 statement: An updated guideline for reporting systematic reviews. *BMJ*, *372*, 10.1136/bmj.n71 PMC8005924 33782057

[CIT0063] Phillips, K. A., Greenberg, J. L., Hoeppner, S. S., Weingarden, H., O'Keefe, S., Keshaviah, A., Schoenfeld, D. A., & Wilhelm, S. (2021). Predictors and moderators of symptom change during cognitive-behavioral therapy or supportive psychotherapy for body dysmorphic disorder. *Journal of Affective Disorders*, *287*, 34–40. 10.1016/j.jad.2021.03.011 33773357 PMC8276884

[CIT0064] Ramnerö, J., & Öst, L.-G. (2004). Prediction of outcome in the behavioural treatment of panic disorder with agoraphobia. *Cognitive Behaviour Therapy*, *33*(*4*), 176–180. 10.1080/16506070410031691 15625791

[CIT0065] Ramnerö, J., & Öst, L.-G. (2007). Therapists’ and clients’ perception of each other and working alliance in the behavioral treatment of panic disorder and agoraphobia. *Psychotherapy Research*, *17*(*3*), 320–328. 10.1080/10503300600650852

[CIT0066] Rosenthal, R. (1995). Writing meta-analytic reviews. *Psychological Bulletin*, *118*(2), 183–192. 10.1037/0033-2909.118.2.183

[CIT0067] Rosmarin, D. H., Bigda-Peyton, J. S., Kertz, S. J., Smith, N., Rauch, S. L., & Björgvinsson, T. (2013). A test of faith in God and treatment: The relationship of belief in God to psychiatric treatment outcomes. *Journal of Affective Disorders*, *146*(*3*), 441–446. 10.1016/j.jad.2012.08.030 23051729

[CIT0068] Samantaray, N. N., Mishra, A., Singh, A. R., Sudhir, P. M., & Singh, P. (2023). Anxiety sensitivity as a predictor, and non-specific therapeutic factors as predictors and mediators of CBT outcome for obsessive-compulsive disorder in a naturalistic mental health setting. *Journal of Affective Disorders*, *324*, 92–101. 10.1016/j.jad.2022.12.085 36584701

[CIT0069] Schmidt, F. L., & Hunter, J. E. (2016). *Methods of meta-analysis: Correcting error and bias in research findings*. SAGE Publications.

[CIT0070] Schünemann, H., Brozek, J., Guyatt, G., & Oxman, A. D. (2013). Grade handbook. Introduction to GRADE Handbook. *The GRADE Working Group*.

[CIT0071] Silva, S., Barbosa, E., Salgado, J., & Cunha, C. (2021). Portuguese validation of the credibility/expectancy questionnaire in routine practice. *Research in Psychotherapy: Psychopathology, Process and Outcome*, *24*(1), 10.4081/ripppo.2021.495 PMC8082527 33937109

[CIT0072] Söchting, I., Tsai, M., & Ogrodniczuk, J. S. (2016). Patients’ perceptions of treatment credibility and their relation to the outcome of group CBT for depression. *Archives of Psychiatry and Psychotherapy*, *18*(4), 10.12740/APP/66485

[CIT0073] Strong, S. R. (1968). Counseling: An interpersonal influence process. *Journal of Counseling Psychology*, *15*(3), 10.1037/h0020229

[CIT0074] Taylor, S. (2003). Outcome Predictors for Three PTSD Treatments: Exposure Therapy, EMDR, and Relaxation Training. *Journal of Cognitive Psychotherapy*, *17*(*2*), 149–162. 10.1891/jcop.17.2.149.57432

[CIT0075] Thomas, B. H., Ciliska, D., Dobbins, M., & Micucci, S. (2004). A process for systematically reviewing the literature: Providing the research evidence for public health nursing interventions. *Worldviews on Evidence-Based Nursing*, *1*(3), 10.1111/j.1524-475X.2004.04006.x 17163895

[CIT0076] Thompson-Hollands, J., Bentley, K. H., Gallagher, M. W., Boswell, J. F., & Barlow, D. H. (2014). Credibility and outcome expectancy in the unified protocol: Relationship to outcomes. *Journal of Experimental Psychopathology*, *5*(1), 72–82. 10.5127/jep.033712

[CIT0077] Thornett, A. M., & Mynors-Wallis, L. M. (2002). Credibility of problem-solving therapy and medication for the treatment of depression among primary care patients. *Medical science monitor : international medical journal of experimental and clinical research*, *8*(3), CR193–CR196. 11887035

[CIT0078] Uebelacker, L. A., Weinstock, L. M., Battle, C. L., Abrantes, A. M., & Miller, I. W. (2018). Treatment credibility, expectancy, and preference: Prediction of treatment engagement and outcome in a randomized clinical trial of hatha yoga vs. health education as adjunct treatments for depression. *Journal of Affective Disorders*, *238*, 10.1016/j.jad.2018.05.009 PMC6901089 29870820

[CIT0079] Viechtbauer, W. (2010). Conducting meta-analysis in R with the metafor package. *Journal of Statistical Software*, *36*(3), 10.18637/jss.v036.i03

[CIT0080] Viechtbauer, W., & Cheung, M. W.-L. (2010). Outlier and influence diagnostics for meta-analysis. *Research Synthesis Methods*, *1*(2), 10.1002/jrsm.11 26061377

[CIT0081] Vos-Vromans, D. C. W. M., Huijnen, I. P. J., Rijnders, L. J. M., Winkens, B., Knottnerus, J. A., & Smeets, R. J. E. M. (2016). Treatment expectations influence the outcome of multidisciplinary rehabilitation treatment in patients with CFS. *Journal of Psychosomatic Research*, *83*, 40–45. 10.1016/j.jpsychores.2016.02.004 27020075

[CIT0082] Wallin, E. E. K., Mattsson, S., & Olsson, E. M. G. (2016). The preference for internet-based psychological interventions by individuals without past or current use of mental health treatment delivered online: A survey study with mixed-methods analysis. *JMIR Mental Health*, 3(2). 10.2196/mental.5324 PMC4925931 27302200

[CIT0083] Wampold, B. E. (2015). How important are the common factors in psychotherapy? An update. *World Psychiatry*, *14*(3), 270–277. 10.1002/wps.20238 26407772 PMC4592639

[CIT0084] Wampold, B. E., Mondin, G. W., Moody, M., Stich, F., Benson, K., & Ahn, H. (1997). A meta-analysis of outcome studies comparing bona fide psychotherapies: Empiricially, “all must have prizes”. *Psychological Bulletin*, *122*(3), 10.1037//0033-2909.122.3.203

[CIT0085] Westra, H. A., Constantino, M. J., Arkowitz, H., & Dozois, D. J. A. (2011). Therapist differences in cognitive-behavioral psychotherapy for generalized anxiety disorder: A pilot study. *Psychotherapy*, *48*(3), 283–292. 10.1037/a0022011 21688930

[CIT0086] Wolff, M. C., & Hayes, J. A. (2009). Therapist variables: Predictors of process in the treatment of alcohol and other drug problems. *Alcoholism Treatment Quarterly*, *27*(1), 51–65. 10.1080/07347320802586791

