# Supplementary material for: Therapist and treatment credibility in treatment outcomes: A systematic review and meta-analysis of clients’ perceptions in individual and face-to-face psychotherapies
Source: Psychother Res. 2024 Jan 4;35(1):139–54. doi: 10.1080/10503307.2023.2298000 (PMC11771474; doi:10.1080/10503307.2023.2298000)
Supplement: tpsr-2023-0236-File005 [file TPSR_A_2298000_SM0709.docx]

**Supplementary Material**

1. **Search Strategy**

***Search Words (for Embase, Medline, PsycInfo):***

1. ((trust adj6 (psychotherap* or therap* or counsel*)) or credib* or ((social or therapist or counsel?or) adj influenc*) or therapist expert*).tw
   AND
2. (psychotherap* or psychodynamic or ((cognitive or behavio*) adj2 (intervention* or therap* or treat*)) or ((brief or early or short-term) adj2 (intervention or therap*)) or counsel* or ((dialectical or interpersonal) adj2 (intervention* or therap* or treat*)) or dissonance based or ((aversion or exposure or implosive or distraction or deactivation) adj therap*) or (acceptance and commitment) or (motivation* adj2 (intervention* or program* or therap*)) or mindfulness or bibliotherap* or ((art or play) adj therap*) or (problem adj (focus or sol*)) or ((support* or nondirective or non-directive or rational emotive) adj therap*) or transactional analysis or trauma focus* or dynamic therapy or systemic therap* or interpersonal therap* or therap* relationship or (mentali* adj2 (therap* or intervention* or treat*))).tw
   AND
3. (outcome* or treatment response or (symptom adj2 (severity or change or improve* or reduction)) or remission or (treatment adj2 effect) or quality of life or ((attitude or behavio?r*) adj (change or improve*))).tw

***Search Words (for Manual Search on Google Scholar):***

The first four pages of every search result obtained from a Google Scholar search were scrutinized using the following search terms:

(Therapist trustworthiness OR therapist expertise OR therapist credibility OR counselor credibility OR counselor influence OR counselor trustworthiness OR counsellor expertise OR treatment credibility) AND treatment outcome

***Search Strategy for Grey Literature:***

The search for dissertations encompassed the British Library’s E-Theses Online Service (EThOS), Open Access Theses and Dissertations (OATD), and the Social Science Research Network (SSRN). In addition, unpublished studies were sought in the National Grey Literature Collection, PsyArxiv, and the National Library of Medicine’s clinical trials repository (clinicaltrials.gov) using the search terms “therapist trustworthiness”, “therapist credibility” or “treatment credibility”. Further, conference abstracts were explored through PsycExtra and Global Health databases on the OVID platform using the main search strategy keywords. Identified potential papers were cross-referenced for related publications. In cases where no related publication was found, subsequent correspondence was initiated with the authors via email.

1. **Studies that were excluded although they appear to meet the inclusion criteria, and the reasons for exclusion**.

| **Study** | **DOI** | **Reason for exclusion** |
| --- | --- | --- |
| Beutler et al. (1975) | 10.1037/h0076326 | Limited statistical information |
| Andersson et al. (2008) | 10.1016/j.chb.2008.02.003 | Using the same dataset with another eligible paper |
| Newman & Fisher (2010) | 10.1521/ijct.2010.3.3.245 | Combined use of credibility/expectancy subscales |
| Altman et al. (2022) | 10.1080/02791072.2021.2020382 | No therapy outcome |
| Gaume et al. (2014) | 10.1111/acer.12469 | No credibility assessment |
| Clark et al. (1999) | 10.1037//0022-006x.67.4.583 | No credibility-outcome analysis |
| Nelson & Borkovec (1989) | 10.1016/0005-7916(89)90048-7 | Using the same dataset with another eligible paper |
| Corrigan & Schmidt (1983) | [10.1037/0022-0167.30.1.64](https://psycnet.apa.org/doi/10.1037/0022-0167.30.1.64) | No therapy outcome |
| Earleywine et al. (2022) | [10.1080/02791072.2021.1912863](https://doi.org/10.1080/02791072.2021.1912863) | No therapy outcome |
| Heesacker (1986) | [10.1037/0022-0167.33.2.107](https://psycnet.apa.org/doi/10.1037/0022-0167.33.2.107) | No treatment |
| Ghaderi et al. (2022) | [10.1017/S1352465821000345](https://doi.org/10.1017/s1352465821000345) | No credibility-outcome analysis |
| Brotto et al. (2020) | 10.1016/j.jsxm.2020.07.080 | Group therapy |
| Koszycki et al. (2022) | [10.1002/cpp.2658](https://doi.org/10.1002/cpp.2658) | No credibility-outcome analysis |
| Borkovec & Nau (1972) | 10.1016/0005-7916(72)90045-6 | No therapy outcome |
| Owen et al. (2019) | 10.1037/ccp0000437 | No credibility assessment |
| Mathiasen et al. (2022) | [10.2196/36577](https://doi.org/10.2196/36577) | No credibility-outcome analyses |
| Haddad et al. (2019) | 10.1002/gps.5279 | Group therapy |
| Goates-Jones & Hill (2008) | 10.1037/0033-3204.45.1.61 | Only session based short term outcome |
| Clark et al. (1994) | 10.1192/bjp.164.6.759 | No credibility-outcome analysis |
| Shepardson et al. (2022) | 10.1037/ser0000622 | No credibility-outcome analysis |
| Beadman et al. (2015) | 10.1016/j.brat.2015.03.013 | No therapy |
| Curtis (1982) | [10.2466/pr0.1981.48.1.12](https://doi.org/10.2466/pr0.1981.48.1.127)7 | No therapy outcome |
| Siegel et al. (2020) | 10.3390/ijerph17114105 | No credibility assessment |
| Ritter et al. (2002) | 10.1080/0959523021000002723 | Group therapy |
| Silverberg, N. D., et al. (2022). | [10.1016/j.apmr.2021.12.005](https://doi.org/10.1016/j.apmr.2021.12.005) | No credibility-outcome analysis |

1. **Sensitivity analyses without missing value imputation for non-significant articles**

Sensitivity analyses were conducted using 21 independent samples and a total of 1374 participants. A comparison of the goodness of fit of a three-level model versus a simpler two-level model was conducted. The results indicated that the three-level model exhibited a better fit to the data, demonstrating an increased capacity to account for the variability observed in the data (X^2^_1_ = 20.93, p < .001). Similar to analyses with imputation, the examination of the relationship between therapist credibility and treatment outcome revealed a statistically significant, albeit small, association (*r* = .19, 95% CI = [.13, .27], *t* = 5.57, *p* < .001). Additionally, a significant level of moderate heterogeneity was observed (*Q* (58) = 94.72, *p* = .002, *I*^2^ = 42.34). The estimated variance components were found to be *τ*^2^_Level 3_ = .01 and *τ*^2^_Level 2_= .00. Further analysis of the heterogeneity indicated that *I*^2^_Level 3_ = 42.34% of the total variation can be attributed to between-study heterogeneity and *I*^2^_Level 2_ = 0% to within-study heterogeneity.

The difference between initial and emergent treatment credibility (*F* (1, 57) = .12, *p* = .73), session-based timing of credibility assessment (*F* (1, 57) = 2.94, *p* = .09), the quality of the papers (*F* (2, 56) = .62, p = .54) and the publication year (*F* (2, 56) = 0.11*, p* = .89) were not found as significant moderators (see Supplementary Table ST2).

**Supplementary Table ST1.**

*Results of the quality assessment*

| References | **SB** | **SD** | **C** | **B** | **DCM** | **W&D** | **G** |
| --- | --- | --- | --- | --- | --- | --- | --- |
| *Therapist Credibility* |  |  |  |  |  |  |  |
| Bathje et al., (2022) | W | M | M | W | W | W | W |
| Farsimadan et al., (2007) | M | M | S | M | S | S | S |
| Gieselmann et al., (2016) | W | S | S | M | S | S | M |
| Grimes & Murdock, (1989) | M | M | W | M | S | W | W |
| Kasarabada et al., (2002) | M | M | W | W | S | S | W |
| LaCrosse, (1980) | M | M | S | M | S | M | S |
| Lawlor et al., (2017) | M | M | W | M | S | M | M |
| Lafferty et al., (1989) | M | M | M | M | S | M | S |
| Ramnerö & Öst, (2007) | M | S | W | M | W | S | W |
|  |  |  |  |  |  |  |  |
| *Treatment Credibility* |  |  |  |  |  |  |  |
| Barlow et al., (1992) | M | S | W | M | W | M | W |
| Barnicot et al., (2019) | M | M | M | W | S | W | W |
| Borkovec & Costello, (1993) | M | S | S | M | S | S | S |
| Borkovec & Mathews, (1988) | M | S | S | M | W | S | M |
| Borkovec et al., (1987) | W | S | W | M | W | M | W |
| Borkovec et al., (2002) | W | S | S | M | S | S | M |
| Carlbring et al., (2005) | W | S | W | M | S | S | W |
| Devilly & Spence, (1999) | W | S | M | M | S | M | M |
| Freeston et al., (1997) | W | S | S | W | S | S | W |
| Greenberg et al., (2019) | W | M | S | W | S | S | W |
| Hardy et al., (1995) | M | S | S | W | S | S | M |
| Harrison et al. (2019) | M | S | S | S | S | M | S |
| Hellström & Öst, (1996) | M | M | S | W | W | S | W |
| Hundt et al., (2014) | W | S | S | W | S | M | W |
| Kim et al., (2015) | M | S | S | W | S | W | W |
| Kuzminskaite et al., (2021) | M | S | M | M | W | M | M |
| Mooney et al., (2014) | W | M | S | S | S | M | M |
| Morrison & Shapiro, (1987) | W | S | S | S | S | S | M |
| Phillips et al., (2021) | M | S | S | M | S | M | S |
| Ramnerö & Öst, (2004) | M | S | M | M | W | S | M |
| Rosmarin et al., (2013) | M | M | S | M | W | W | W |
| Samantaray et al., (2023) | W | M | W | W | S | W | W |
| Taylor, (2003) | W | S | W | M | S | M | W |
| Thompson-Hollands et al., (2014) | M | S | W | W | S | S | W |
| Thornett & Mynors-Wallis, (2002) | M | S | W | M | S | M | M |
| Vos-Vromans et al., (2016) | M | S | S | W | S | S | M |
| Westra, et al., (2011) | W | S | W | M | S | M | W |

*Note*. SB, Selection Bias; SD, Study Design ; C, Confounders; B, Blinding; DCM; Data Collection Method; W&D, Withdrawals and Dropouts; G, Global Quality Assessment; W, Weak; M, Moderate; S, Strong.

**Supplementary Figure SF1.**

Forest plot of the association between therapist credibility and treatment outcome


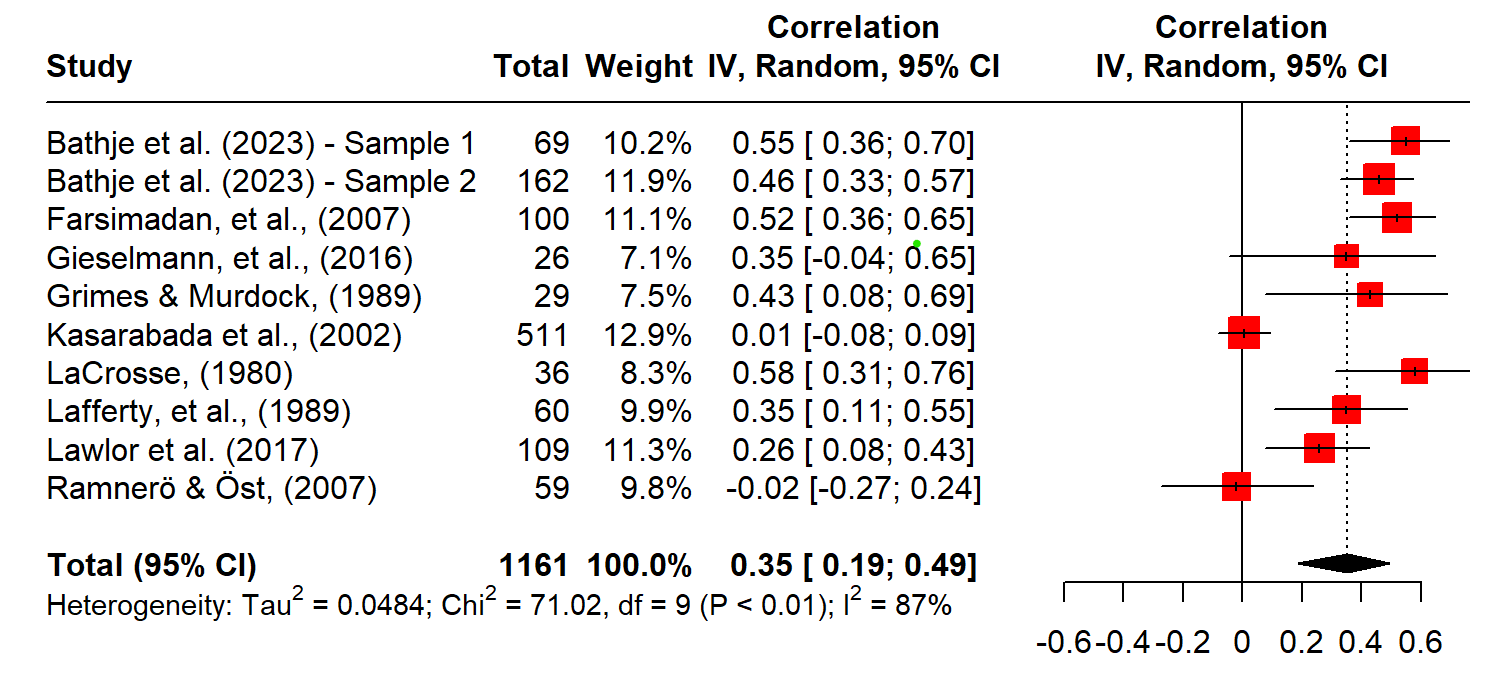


**Supplementary Figure SF2**

*Forest plot of the association between treatment credibility and treatment outcome*

***
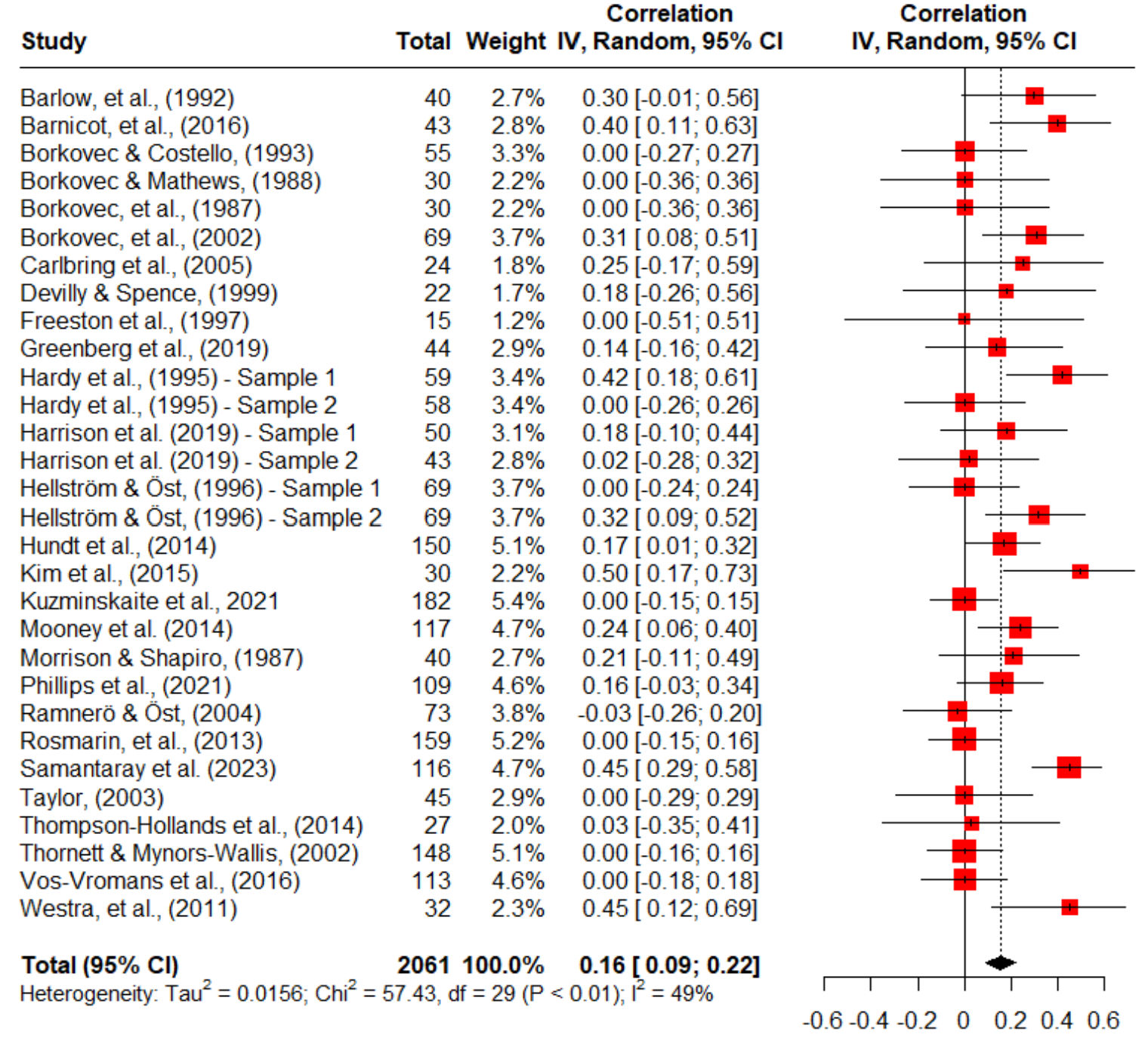
***

**Supplementary Table ST2.**

*Results for the moderator analyses of the three-level meta-analysis without missing value imputation*

| Analysis | No. of independent samples | *r* (SE) | 95% CIs | *p* |
| --- | --- | --- | --- | --- |
| Moderators |  |  |  |  |
| *Timing* |  |  |  |  |
| Initial | 12 | .19 (.04) | .10, .28 | .00 |
| Emergent | 12 | .21 (.05) | -.09, .12 | .73 |
| *Quality Assessment* |  |  |  |  |
| Weak | 10 | .23 (.05) | .13, .33 | .001 |
| Moderate | 8 | .16 (.08) | -.22, .08 | .36 |
| Strong | 3 | .13 (.12) | -.33, .13 | .39 |
| *Publication Year* |  |  |  |  |
| Before 2000 | 6 | .20 (.07) | .06, .33 | .001 |
| 2001-2011 | 3 | .15 (.12) | -.30, .20 | .20 |
| 2012-2023 | 12 | .21 (.08) | -.16, .17 | .17 |

**PRISMA Checklist**

| **Section and Topic** | **Item #** | **Checklist item** | **Location where item is reported** |
| --- | --- | --- | --- |
| **TITLE** | | |  |
| Title | 1 | Identify the report as a systematic review. | Title – page 1 |
| **ABSTRACT** | | |  |
| Abstract | 2 | See the PRISMA 2020 for Abstracts checklist. | Page 1 |
| **INTRODUCTION** | | |  |
| Rationale | 3 | Describe the rationale for the review in the context of existing knowledge. | Pages 2-6 |
| Objectives | 4 | Provide an explicit statement of the objective(s) or question(s) the review addresses. | Pages 5-6 |
| **METHODS** | | |  |
| Eligibility criteria | 5 | Specify the inclusion and exclusion criteria for the review and how studies were grouped for the syntheses. | Page 6-7; Groups were identified in page 8 |
| Information sources | 6 | Specify all databases, registers, websites, organisations, reference lists and other sources searched or consulted to identify studies. Specify the date when each source was last searched or consulted. | Page 6 |
| Search strategy | 7 | Present the full search strategies for all databases, registers and websites, including any filters and limits used. | Page 6 & Supplementary Material Section 1 |
| Selection process | 8 | Specify the methods used to decide whether a study met the inclusion criteria of the review, including how many reviewers screened each record and each report retrieved, whether they worked independently, and if applicable, details of automation tools used in the process. | Page 6 |
| Data collection process | 9 | Specify the methods used to collect data from reports, including how many reviewers collected data from each report, whether they worked independently, any processes for obtaining or confirming data from study investigators, and if applicable, details of automation tools used in the process. | Page 8 |
| Data items | 10a | List and define all outcomes for which data were sought. Specify whether all results that were compatible with each outcome domain in each study were sought (e.g. for all measures, time points, analyses), and if not, the methods used to decide which results to collect. | Page 8 |
|  | 10b | List and define all other variables for which data were sought (e.g. participant and intervention characteristics, funding sources). Describe any assumptions made about any missing or unclear information. | Page 6 & 8 |
| Study risk of bias assessment | 11 | Specify the methods used to assess risk of bias in the included studies, including details of the tool(s) used, how many reviewers assessed each study and whether they worked independently, and if applicable, details of automation tools used in the process. | Page 9 & Supplementary Material Table 1 |
| Effect measures | 12 | Specify for each outcome the effect measure(s) (e.g. risk ratio, mean difference) used in the synthesis or presentation of results. | Page 9 |
| Synthesis methods | 13a | Describe the processes used to decide which studies were eligible for each synthesis (e.g. tabulating the study intervention characteristics and comparing against the planned groups for each synthesis (item #5)). | Page 8 |
|  | 13b | Describe any methods required to prepare the data for presentation or synthesis, such as handling of missing summary statistics, or data conversions. | Page 10 |
|  | 13c | Describe any methods used to tabulate or visually display results of individual studies and syntheses. | Page 10 |
|  | 13d | Describe any methods used to synthesize results and provide a rationale for the choice(s). If meta-analysis was performed, describe the model(s), method(s) to identify the presence and extent of statistical heterogeneity, and software package(s) used. | Page 11 |
|  | 13e | Describe any methods used to explore possible causes of heterogeneity among study results (e.g. subgroup analysis, meta-regression). | Page 11 |
|  | 13f | Describe any sensitivity analyses conducted to assess robustness of the synthesized results. | Page 9 |
| Reporting bias assessment | 14 | Describe any methods used to assess risk of bias due to missing results in a synthesis (arising from reporting biases). | Page 11 |
| Certainty assessment | 15 | Describe any methods used to assess certainty (or confidence) in the body of evidence for an outcome. | Page 9 |
| **RESULTS** | | |  |
| Study selection | 16a | Describe the results of the search and selection process, from the number of records identified in the search to the number of studies included in the review, ideally using a flow diagram. | Page 12 & Figure 1 |
|  | 16b | Cite studies that might appear to meet the inclusion criteria, but which were excluded, and explain why they were excluded. | Supplementary Material |
| Study characteristics | 17 | Cite each included study and present its characteristics. | Tables 1 and 2 |
| Risk of bias in studies | 18 | Present assessments of risk of bias for each included study. | Supplementary Material Table 1 |
| Results of individual studies | 19 | For all outcomes, present, for each study: (a) summary statistics for each group (where appropriate) and (b) an effect estimate and its precision (e.g. confidence/credible interval), ideally using structured tables or plots. | Tables 1 and2 & Supplementary Material Figures 1 and 2 |
| Results of syntheses | 20a | For each synthesis, briefly summarise the characteristics and risk of bias among contributing studies. | Pages 12-13 |
|  | 20b | Present results of all statistical syntheses conducted. If meta-analysis was done, present for each the summary estimate and its precision (e.g. confidence/credible interval) and measures of statistical heterogeneity. If comparing groups, describe the direction of the effect. | Pages 12-17 |
|  | 20c | Present results of all investigations of possible causes of heterogeneity among study results. | Pages 12-17 |
|  | 20d | Present results of all sensitivity analyses conducted to assess the robustness of the synthesized results. | Pages 13 & 17 |
| Reporting biases | 21 | Present assessments of risk of bias due to missing results (arising from reporting biases) for each synthesis assessed. | Pages 14 & 15, Supplementary Material |
| Certainty of evidence | 22 | Present assessments of certainty (or confidence) in the body of evidence for each outcome assessed. | Pages 14 & 16 |
| **DISCUSSION** | | |  |
| Discussion | 23a | Provide a general interpretation of the results in the context of other evidence. | Page 17-21 |
|  | 23b | Discuss any limitations of the evidence included in the review. | Page 19 |
|  | 23c | Discuss any limitations of the review processes used. | Page 21-22 |
|  | 23d | Discuss implications of the results for practice, policy, and future research. | Page 22-23 |
| **OTHER INFORMATION** | | |  |
| Registration and protocol | 24a | Provide registration information for the review, including register name and registration number, or state that the review was not registered. | Page 6 |
|  | 24b | Indicate where the review protocol can be accessed, or state that a protocol was not prepared. | Page 6 |
|  | 24c | Describe and explain any amendments to information provided at registration or in the protocol. | Page 6 |
| Support | 25 | Describe sources of financial or non-financial support for the review, and the role of the funders or sponsors in the review. | Title page – Page 1 |
| Competing interests | 26 | Declare any competing interests of review authors. | Title page – Page 1 |
| Availability of data, code and other materials | 27 | Report which of the following are publicly available and where they can be found: template data collection forms; data extracted from included studies; data used for all analyses; analytic code; any other materials used in the review. | Page 12 |
